# Structural Evolution of AlN Nanoclusters and the Elemental Chemisorption Characteristics: Atomistic Insight

**DOI:** 10.3390/nano9101420

**Published:** 2019-10-04

**Authors:** Xi Nie, Zhao Qian, Wenzheng Du, Zhansheng Lu, Hu Li, Rajeev Ahuja, Xiangfa Liu

**Affiliations:** 1 Key Laboratory for Liquid-Solid Structural Evolution and Processing of Materials (Ministry of Education), School of Materials Science and Engineering, Shandong University, Jinan 250061, China; 17861412028@139.com (X.N.); 13127150302@163.com (W.D.); xfliu@sdu.edu.cn (X.L.); 2 School of Materials Science and Engineering, Henan Normal University, Xinxiang 453007, China; zslu@henannu.edu.cn; 3 School of Electrical and Electronic Engineering, University of Manchester, Manchester M139PL, UK; Hu.Li@manchester.ac.uk; 4 Condensed Matter Theory Group, Department of Physics and Astronomy, Ångström Laboratory, Uppsala University, 75120 Uppsala, Sweden; rajeev.ahuja@physics.uu.se

**Keywords:** AlN, low-dimensional material, atomic cluster, electronic structure, HSE06 hybrid functional

## Abstract

A theoretical insight into the structural evolution of AlN atomic clusters and the chemisorption of several common alloying elements on a large cluster has been performed in the framework of state-of-the-art density functional theory calculations. We report the findings that the longitudinal growth takes precedence during the early stage of structural evolution of small AlN clusters, when the longitudinal dimension becomes stable, the AlN cluster proceeds with cross-growth and blossoms into the large-size Al_60_N_60_. Upon the growth of clusters, the structures tend to become well-knit gradually. As for the evolution of electronic structures of AlN clusters through the HSE06 calculations, the density of states curves become more and more nondiscrete with the atomic structures evolving from small to large size and tend to resemble that of the Wurtzite AlN. The chemisorption characteristics of the large Al_60_N_60_ cluster towards different elements such as Al, N, Fe and Cu are also theoretically unveiled, in which it is interestingly found that the N and Cu atoms are likely to be adsorbed similarly at the growth edge position of the Al_60_N_60_ cluster and the density of states curves of these two chemisorption systems near the Fermi level also show some interesting similarities.

## 1. Introduction

Atomic clusters play important roles in the nucleation of solid phases. It is worthwhile to perform fundamental research on clusters to reveal their structures and properties at the atomic and electronic levels. Li et al. systematically investigated the structural evolution of gold–germanium bimetallic clusters and the nonlinear optical properties, chemical properties of a series of alkali-metals-adsorbed gold–germanium bimetallic clusters, in which it was found that the atomic structure of gold–germanium bimetallic clusters with adsorbed alkali metals did not change significantly and alkali metals tended to adhere to the surface or edge of clusters [1]. Yan et al. studied the structural evolution of as clusters using the first principles method [2]. Die et al. investigated the structural and magnetic properties of Cu4M clusters and found that in the most stable Cu4M clusters, the positions of M atoms are the most coordinated [3]. In recent years, the light-element aluminum-based clusters have received extensive attention and have been studied [4,5,6], especially the aluminum–pnictogen system. Aluminum nitride (AlN) is one example, which has many desirable properties, such as high thermal conductivity, high temperature resistance, impact resistance and a low expansion coefficient [7]. AlN can be prepared by the reaction of aluminum salt with ammonia, chemical vapor deposition, etc. [8,9,10]. In vacuum, researchers have used magnetron reactive sputtering technology to make the sputtered aluminum react with nitrogen to prepare a new AlN nanofilm and Kishimoto et al. [11] studied AlN film growth on sapphire in an experiment. In addition, in the magnetron reactive sputtering experiment, a series of Al_n_N_m_ clusters were also observed [12]. Meanwhile, some researchers used the ab initio methods to study the aluminum–nitrogen system [13,14,15,16]. Saeedi et al. performed density functional theory to calculate the electronic properties of octahedral Al_n_N_n_ cages and Al_n_P_n_ cages to discuss the isotropic chemical shielding parameters of Al_n_N_n_ cages or Al_n_P_n_ cages in different electrostatic environments [17]. BelBruno designed the structures of Al_n_N_n_ (n = 2–4) clusters using density functional theory and compared them with the carbon and boron nitride clusters [18]. Furthermore, some scholars have studied the hydrogen storage and gas detecting properties of AlN clusters [19,20]. However, there are few reports on the electronic structures’ evolution and growth of AlN clusters employing the hybrid functionals, although the growth of aluminum nitride has been reported in experiments concerning the preparation of AlN thin films, blocks or single crystals [21,22,23,24].

In this article, we have theoretically investigated the structural evolution concerning the growth of AlN clusters from the perspective of atomic and electronic structures using the HSE06 hybrid functional, along with the characteristics of the bond lengths and energetics of AlN clusters. In order to understand the chemisorption of common alloying elements such as Fe, Cu, Al, N on AlN clusters, we also simulated the interaction between these elements and the Al_60_N_60_ large clusters to unveil their chemisorption characteristics. In the previous experimental study, we found that copper can be observed on the AlN_p_/Al interface by adding copper powder to AlN_p_-reinforced Al composites. In addition, when the Fe power is added to the AlN_p_-reinforced Al composites, it is not aggregated at the interface but dispersedly distributed in the composite material [25]. This work is thus proposed to help understand the experimental result and expose structures and chemisorption properties of AlN atomic clusters, as well as providing a deep theoretical guidance for experimentalists.

## 2. Methods

In this study, the calculations of total energies, atomic forces, and structure optimizations have been performed using the generalized gradient approximation (GGA) [26] in the form of Perdew–Burke–Ernzerhof (PBE) based on the density functional theory (DFT) [27,28]. The projector augmented wave (PAW) [29] method has been employed in the Vienna Ab initio Simulation Package [30]. Considering that the experimental exposed surfaces of AlN are usually made up of (10$\bar{1}$0) and (000$\bar{1}$) [31], we have established the models in the supercell of Wurtzite AlN through cutting out the smallest structural unit of the hexagonal prism (Al_6_N_6_ cluster). In order to avoid interaction between clusters in the x, y and z directions, all the models are separated by the vacuum space of 20 Å. The K-points mesh of 1×1×1 has been used within the Monkhorst–Pack scheme in the stage of geometry optimizations and electronic structure calculations. The plane-wave energy cut-off of 520 eV has been employed for all the structure optimizations. We used a conjugate gradient algorithm to perform the structure optimizations and relax all ionic positions until the force on each ion is lower than 0.02 eV/Å. In order to calculate the electronic structure of the clusters and their chemisorption systems more accurately, we have considered the effects of nonlocal exchange and used the screened hybrid functional of Heyd, Scuseria, and Ernzerhof (HSE06) [32]. In HSE06, only the local part of the exact exchange energy is treated by Hartree-Fork theory, while the remaining part is treated by DFT. In our paper, the screening parameter μ was set to 0.2, conforming to the HSE06 functional.

## 3. Results and Discussion

### 3.1. Structural Evolution of AlN Clusters

**Figure 1:**
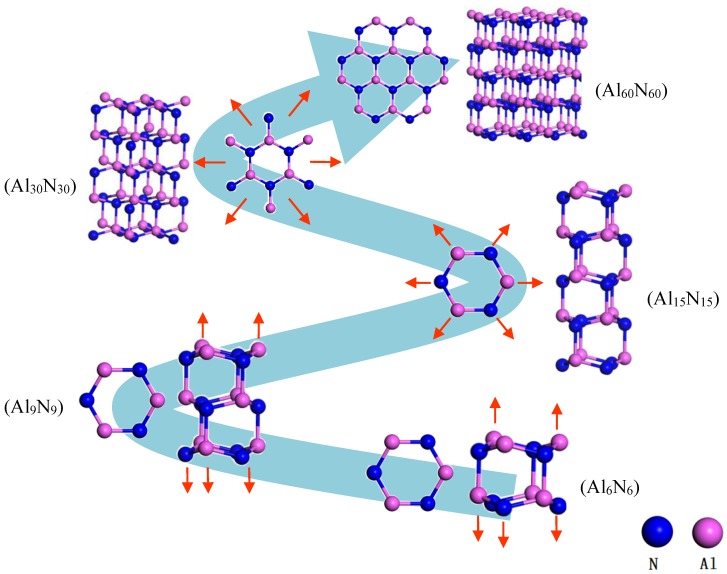
Outline of the stepwise formation (both the top view and the side view) of the Al_60_N_60_ large clusters. The red arrows stand for the growth direction of each respective cluster, and the big blue arrow stands for the overall evolution of clusters from small to large.

During the structural evolution and growth of AlN clusters, the five most representative clusters are selected. The smallest cluster unit is Al_6_N_6_. When the Al_6_N_6_ cluster first grows longitudinally into a stable two-layer hexagonal prism structure, the representative cluster is Al_9_N_9_. For the Al_15_N_15_ cluster, it is formed when the longitudinal dimension of the cluster becomes stable. The Al_15_N_15_ cluster then evolves to Al_30_N_30_ in the transverse direction after the cluster size is stabilized in the longitudinal direction. The Al_60_N_60_ is a large cluster structure evolved from Al_30_N_30_ with continued transverse growth. The structural evolution process of AlN clusters in the atomic scale is shown in Figure 1. The small AlN unit cluster prefers growing along the longitudinal direction in the beginning, after which, the larger cluster would continue growing along the transverse direction when the longitudinal growth reaches stability. For the Al_15_N_15_ cluster, each aluminum or nitrogen atom of the hexatomic ring connects with a nitrogen or aluminum atom to lead to the formation of the Al_30_N_30_ cluster. The dangling aluminum and nitrogen atoms in the Al_30_N_30_ cluster would further absorb atoms, forming complete hexatomic rings and growing into a large aluminum nitride cluster (Al_60_N_60_ cluster).

**Figure 2:**
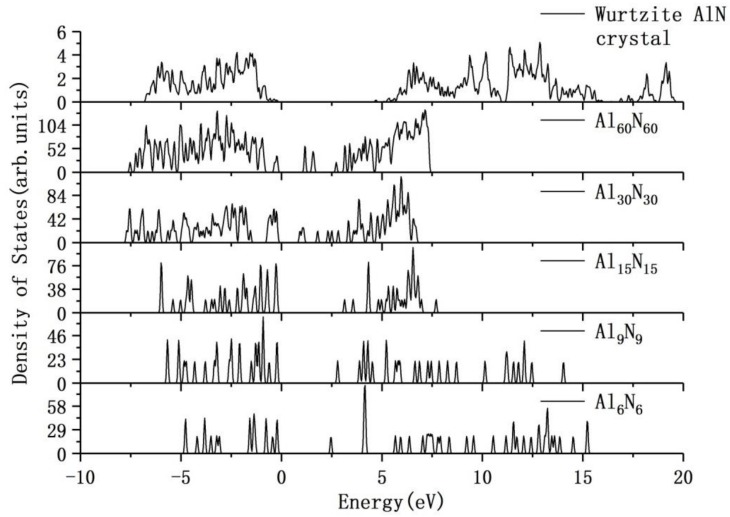
The electronic density of states of the Al_6_N_6_, Al_9_N_9_, Al_15_N_15_, Al_30_N_30_, Al_60_N_60_ atomic clusters and the WurtziteAlN crystal through the HSE06 calculations (the Fermi level is set at zero).

In addition to the evolution of AlN clusters in atomic structures, we have also investigated the evolution of AlN clusters in electronic structures. In order to obtain the more accurate electronic structure, we have employed the HSE06 hybrid functional in calculations. The electronic structures of the Al_6_N_6_, Al_9_N_9_, Al_15_N_15_, Al_30_N_30_ and Al_60_N_60_ clusters are compared with the Wurtzite AlN crystal, which can be shown in Figure 2. The Wurtzite AlN crystal has the continuous curve of density of states. From the figure, it is obvious that the small AlN clusters, such as Al_6_N_6_, Al_9_N_9_ and Al_15_N_15_, have the discrete density of states curves. With the evolution and growth of AlN clusters, the density of states curves tend to be continuous and become more similar to the DOS curve of the Wurtzite AlN crystal gradually. For the larger Al_30_N_30_ and Al_60_N_60_ atomic clusters, the density of states curves show four and two deep levels, respectively, in the band gap through the HSE06 calculations. Near the Fermi level, in accordance with the increase of AlN clusters, the HOMO–LUMO gap is also gradually increased.

**Figure 3:**
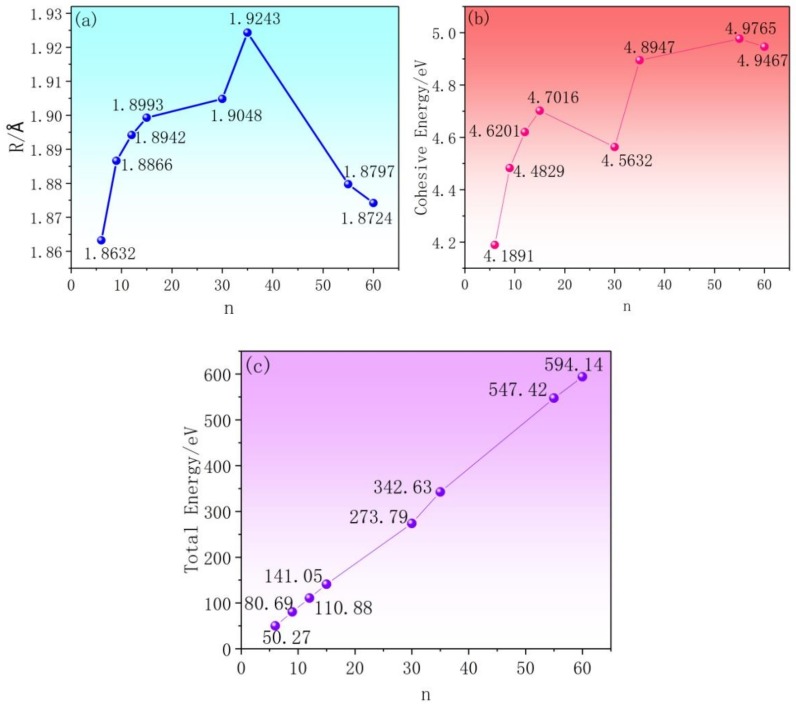
(**a**) The average bond lengths, (**b**) the cohesive energies and (**c**) the total energies of the (AlN)_n_ (n = 6,9,12,15,30,35,55,60) clusters.

In order to further investigate the properties of AlN clusters, the average bond length, cohesive energy and total energy changes during the structural evolution of atomic clusters have been analyzed, which can be seen in Figure 3. Figure 3a shows the average Al-N bond lengths of (AlN)_n_ clusters: with the growth and evolution of clusters, the average bond length gradually increases; when the cluster reaches a certain size, the bond length attains the maximum value. After that, the average bond length begins to decrease and the large cluster tends to shrink. Figure 3b provides the cohesive energies of (AlN)_n_ clusters, which are calculated using the previous method [33,34] based on the formula as follows:

E_coh_ = −[E(AlN)_n_ − nE(Al) − nE(N)]/2n (1)

where E(AlN)_n_ is the total energy of the investigated (AlN)_n_ (n = 6,9,12,15,30,35,55,60) cluster system, while E_Al_ and E_N_ stand for the total energies of a single aluminum atom and nitrogen atom, respectively. When the AlN clusters grow in the early stage, the cohesive energies tend to increase. While, the cohesive energy decreases rapidly when n = 30, which is related to the structure of the Al_30_N_30_ cluster. Each atom in the hexatomic ring of the Al_30_N_30_ cluster is connected with a hetero-atom, which dangles outside and results in a sudden decrease in cohesive energy. After those dangling bonds are saturated by growth, the cohesive energy of the cluster would increase again. Figure 3c shows us the total energies of the investigated clusters, from which it can be seen that with the growth and evolution of the clusters, the total energy increases almost linearly.

### 3.2. Chemisorption of Al, N, Fe, Cu Atoms on the Al_60_N_60_ Large Cluster

As is known, the large clusters especially, in short- or medium-range order could play important roles in nucleation of the corresponding solid phase in materials science. Take the in-situ AlN-reinforced Al alloys for example, Cu is a common strengthening element in aluminum alloys, which can significantly increase strength by precipitation hardening; Fe is also an important element in rapidly cooled aluminum alloys. For chemisorptions of Al and N atoms, it is useful to understand the evolution and growth of AlN clusters. It is essential to study the interactions between the AlN cluster and the alloying elements, which can help to understand the distribution of those alloying elements in solidified materials. In this work, the fundamental interactions between several common alloying elements (Al, N, Fe, Cu) and the Al_60_N_60_ large cluster are investigated considering different chemisorption sites. There are three different positions on the Al_60_N_60_ large cluster: top, edge and side. Due to the existence of equivalent sites, two different sites are studied for the top position: one above the center of the six rings and the other above the Al-N bond. There are four different sites for the edge position: near the N atom with four bonds, near the Al atom with three bonds, near the N atom with three bonds and near the Al atom with two bonds. There are two different sites for the side position: one is near the recessed Al atom, and the other is near the Al-N bond. The adsorption energy E_ad_ is calculated as follows:

E_ad_ = E_Al60N60+M_ − E_Al60N60_ − E_M_ (2)

where E_Al60N60+M_ is the total energy of the system with M element (Al, N, Fe or Cu) adsorbed on the Al_60_N_60_ cluster, while E_Al60N60_ and E_M_ stand for the total energies of the Al_60_N_60_ cluster and the isolated M (Al, N, Fe or Cu) atom, respectively. We considered the chemisorption of Al, N, Fe and Cu at different positions (top, side and edge) of the Al_60_N_60_ large cluster. A total of eight chemisorption sites for every elemental sorption are studied. After calculations, we have chosen the respective site with the largest adsorption energy for each position (i.e., the adsorption energies described below are the largest for the corresponding positions) in our research.

**Figure 4:**
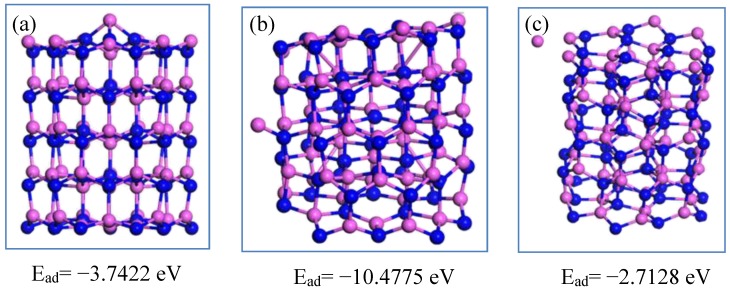
The chemisorption of Al at different sites of the Al_60_N_60_ cluster. The Al and N atoms are shown in pink and blue. The Al atom is located at the top, side and edge position of the Al_60_N_60_ cluster shown in (**a**–**c**), respectively.

**Figure 5:**
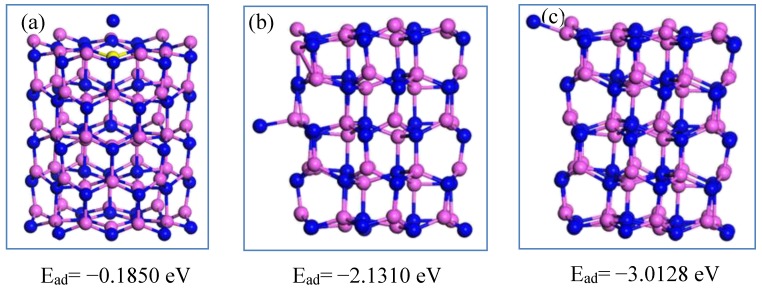
The chemisorption of N element at different sites of the Al_60_N_60_ cluster. The Al and N atoms are shown in pink and blue. The N atom is located at the top, side and edge position of the Al_60_N_60_ cluster shown in (**a**–**c**), respectively.

The chemisorption characteristics towards the Al atom are shown in Figure 4. When the Al atom is adsorbed at the side of the Al_60_N_60_ large cluster, the E_ad_ is the maximum of 10.4775 eV, almost three times those at the top or edge positions. Thus, the aluminum is likely to adsorb and accumulate at the side of the Al_60_N_60_ cluster. Figure 5 shows the chemisorption of nitrogen. When the nitrogen atom is adsorbed at the edge position, the E_ad_ is the maximum of 3.0128 eV. Thus, the nitrogen is more likely to be adsorbed at the edge of the growth frontier of AlN. In the Al melt, the growth atmosphere of AlN is rich in aluminum atoms and poor in nitrogen atoms, so the growth of AlN mainly depends on the deposition/adsorption of nitrogen atoms on AlN. As shown in Figure 5, the nitrogen atoms are likely to adsorb and accumulate at the edge site instead of at the top or side sites of the cluster, which makes AlN tend to grow incliningly instead of longitudinally or transversely. This theoretical finding may provide some guidance for the growth of AlN.

**Figure 6:**
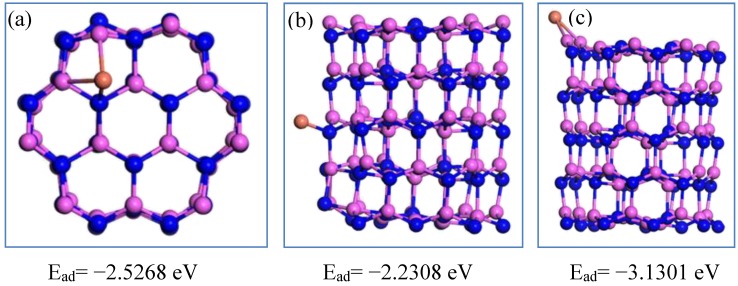
The chemisorption of Cu at different sites of the Al_60_N_60_ cluster. The Al, N and Cu atoms are shown in pink, blue and orange. The Cu atom is located at the top, side and edge position of Al_60_N_60_ cluster shown in (**a**) (top view), (**b**,**c**), respectively.

**Figure 7:**
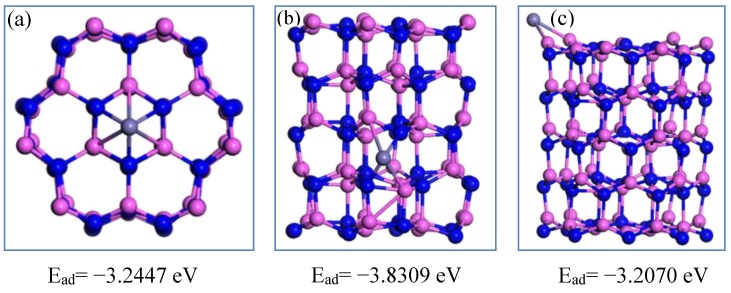
The chemisorption of Fe at different sites of the Al_60_N_60_ cluster. The Al, N and Fe atoms are shown in pink, blue and gray. The Fe atom is located at the top, side and edge position of Al_60_N_60_ cluster shown in (**a**) (top view), (**b**,**c**), respectively.

The chemisorption of Cu atoms on the Al_60_N_60_ large cluster at different positions is shown in Figure 6. In the Al_60_N_60_ cluster, compared with the top and side sites, the copper atom has the highest adsorption energy at the edge site. It can be seen that copper is more likely to be adsorbed at the edge site of the cluster, which is similar to the stacking mode of nitrogen atoms. The chemisorption of iron atoms at different positions of the Al_60_N_60_ cluster is shown in Figure 7. Compared with the top and edge positions, the E_ad_ of Fe element at the side is the largest. It is shown that the Fe atom is easier to accumulate at the side of the Al_60_N_60_ cluster.

**Figure 8:**
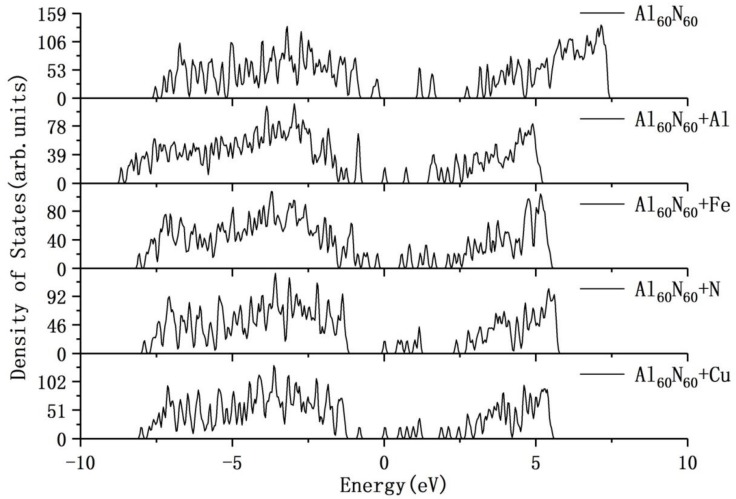
The electronic density of states of the pristine Al_60_N_60_ cluster and the cluster adsorbing Al, N, Cu and Fe atoms, respectively, through the HSE06 calculations (Fermi energy is set to zero).

In order to discuss the effects of Al, N, Fe and Cu atoms on the electronic properties of the Al_60_N_60_ cluster, we have also investigated the electronic structures based on the configurations corresponding to the respective chemisorption systems with the largest adsorption energies of Al, N, Fe and Cu atoms on the cluster through the HSE06 calculations (shown in Figure 8). After Al or Fe atomic chemisorption, the electronic band gap of the system decreases, which illustrates that the Al_60_N_60_ large cluster is sensitive to the atomic chemisorption. What is more, the electronic density of states curves of the N-chemisorption and Cu-chemisorption systems near the Fermi level show some interesting similarities. This corresponds with the above absorption site similarity of the two systems. After adsorbing N or Cu atoms, a small number of deep levels are produced between the HOMO and LUMO.

## 4. Summary and Outlook

In summary, the AlN clusters prefer to grow longitudinally in the early stage and then evolve to the large cluster transversely by using a first principles method. During the structural evolution process, the cohesive energy generally increases and the large-size AlN clusters tend to shrink. The elemental chemisorption studies show that the copper and nitrogen atoms have similar chemisorption characteristics on the Al_60_N_60_ large cluster. It is also found that the electronic density of states curves of the N-chemisorption and Cu-chemisorption systems near the Fermi level show some interesting similarities through HSE06 calculations. This work is proposed to provide valuable theoretical clues and ab initio analyses for the experimentalists in the field and help to deepen understanding of the broader aluminum–pnictogen system at the basic atomic and electronic levels.
